# Fast-track development of an *in vitro* 3D lung/immune cell model to study *Aspergillus* infections

**DOI:** 10.1038/s41598-017-11271-4

**Published:** 2017-09-14

**Authors:** P. Chandorkar, W. Posch, V. Zaderer, M. Blatzer, M. Steger, C. G. Ammann, U. Binder, M. Hermann, P. Hörtnagl, C. Lass-Flörl, D. Wilflingseder

**Affiliations:** 1 Division of Hygiene and Medical Microbiology, Medical University of Innsbruck, Innsbruck, Austria; 2 Experimental Orthopedics, Medical University of Innsbruck, Innsbruck, Austria; 3 Department of Anesthesiology and Critical Care Medicine, Medical University Innsbruck, Innsbruck, Austria; 4 Central Institute for Blood Transfusion & Immunological Department, Medical University of Innsbruck, Innsbruck, Austria

## Abstract

To study interactions of airborne pathogens, e.g. *Aspergillus* (*A.) fumigatus* with upper and lower respiratory tract epithelial and immune cells, we set up a perfused 3D human bronchial and small airway epithelial cell system. Culturing of normal human bronchial or small airway epithelial (NHBE, SAE) cells under air liquid interphase (ALI) and perfusion resulted in a significantly accelerated development of the lung epithelia associated with higher ciliogenesis, cilia movement, mucus-production and improved barrier function compared to growth under static conditions. Following the accelerated differentiation under perfusion, epithelial cells were transferred into static conditions and antigen-presenting cells (APCs) added to study their functionality upon infection with *A. fumigatus*. Fungi were efficiently sensed by apically applied macrophages or basolaterally adhered dendritic cells (DCs), as illustrated by phagocytosis, maturation and migration characteristics. We illustrate here that perfusion greatly improves differentiation of primary epithelial cells *in vitro*, which enables fast-track addition of primary immune cells and significant shortening of experimental procedures. Additionally, co-cultured primary DCs and macrophages were fully functional and fulfilled their tasks of sensing and sampling fungal pathogens present at the apical surface of epithelial cells, thereby promoting novel possibilities to study airborne infections under conditions mimicking the *in vivo* situation.

## Introduction

Understanding the process of attachment of inhaled pathogens to highly differentiated epithelial cells, immune cell transmigration through respiratory epithelia and the removal of airborne particles by DCs or macrophages in a spatiotemporal manner proves to be difficult *in vivo* and *in vitro* due to lack of appropriate tools. Complex 3D *in vitro* systems consisting of airway epithelia, immune cells and airborne particles comprise valuable tools to characterize host-pathogen interactions in respiratory tissues - different approaches to design highly sophisticated *in vitro* systems are currently under development, but are often lacking the immune component^1–3^. Therefore, we set up here a novel design of epithelial/immune cell co-cultures comprising growth of primary epithelial cells under perfusion prior to addition of primary DCs or macrophages, which accelerated the experimental procedure by more than two weeks. DCs and macrophages were further analysed for their functionality after infection of the co-culture system with the airborne pathogen *Aspergillus fumigatus. A. fumigatus* produces thousands of conidia - 2–3 µm in size, which become airborne and can affect both upper and lower respiratory tracts^4, 5^. This is also the reason why we set up perfused *in vitro* systems comprising either normal human bronchial (upper respiratory tract) or small airway (lower respiratory tract) epithelial cells. In healthy individuals, the airway epithelium is able to efficiently clear fungal conidia through mucociliary mechanisms as well as activation of immunological mechanisms^6–8^. Production of cytokines and chemokines by airway epithelial cells results in recruitment of neutrophils, alveolar macrophages and DCs to the sites of infection, which in turn influence adaptive immunity^9, 10^.

DCs are the most potent antigen presenting cells in the respiratory mucosa and upon sampling antigens, DCs mature and migrate to the proximate lymph node, where they prime and polarize CD4^+^ T helper (Th) cell responses^11–15^. In the case of allergens, DCs mainly mediate Th2 polarization, which in turn drives an immunoglobulin E (IgE) response from B cells. *Aspergillus*-related diseases are classified as invasive and chronic pulmonary aspergillosis or allergic bronchopulmonary aspergillosis (ABPA)^16, 17^. Invasive pulmonary aspergillosis (IPA) is mainly found in individuals undergoing chemotherapy or under corticosteroid therapy, and also in human immunodeficiency (HIV)-1-positive individuals^18, 19^. ABPA is especially found in individuals with predisposing lung diseases, i.e. asthma or cystic fibrosis, and is caused by persistence of fungal spores in pulmonary tissues thereby allowing germination due to inefficient clearance by immune cells^9, 20^.

To study infections of the upper (ABPA) and lower (IPA) respiratory tract and to provide a 3D cell culture model that allows the fast investigation of epithelial permeability and immune cell reactions in a realistic and easy to handle *in vitro* system, we set up a perfused three-dimensional cell culture model. Such perfused and highly differentiated epithelia were then used to attach myeloid DCs to the basolateral or macrophages to the apical side and within this system DC and macrophage functions, i.e. DC maturation and migration or macrophage attraction and phagocytosis, were analysed in a three-dimensional space after fungal infection.

## Results

### Perfused dynamic culture conditions exhibit a superior performance in terms of airway cell development

**Figure 1:**
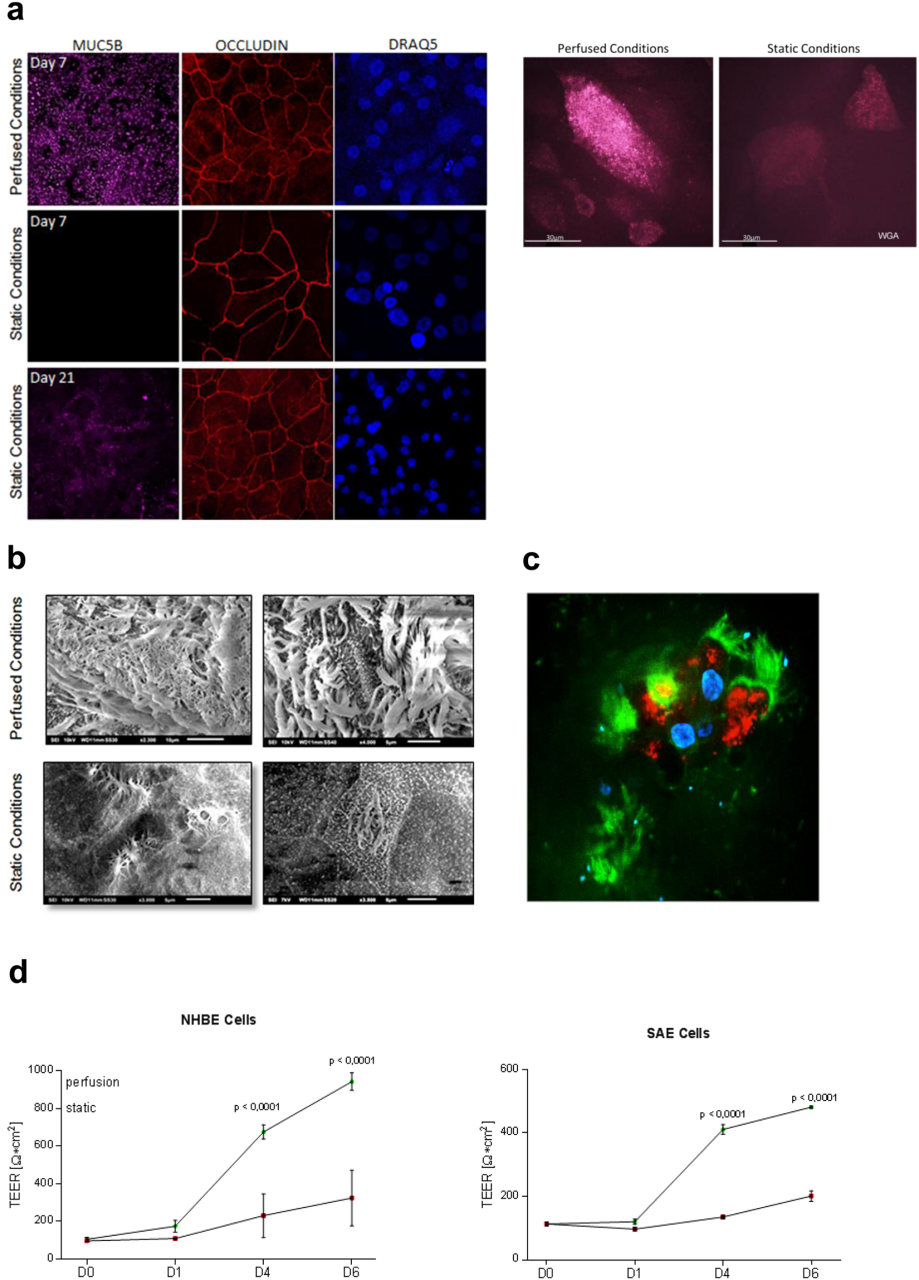
Superior growth and membrane integrity of respiratory cells in ALI under perfused conditions. ((**a**) left) NHBE cells cultured in a dynamic perfused system (upper panel) were fully differentiated on day 7 in ALI - they illustrated high amounts of mucus production (lilac), and well-developed tight junctions (red), while under static conditions no mucus was produced at all on day 7 (middle panel). Mucus production under static conditions started around day 21 and at this time also well differentiated tight junctions were formed (lower panel). Nuclei were stained using Draq5 (blue). ((**a**) right) Cilia were stained on life cells grown under perfused (left) or static (right) conditions using wheat-germ agglutinin (WGA). Also these analyses revealed higher ciliogenesis in perfused settings. (**b**) The higher differentiation of NHBE cells grown under perfusion (upper panel) compared to static conditions (lower panel) on day 7 post ALI was also illustrated by SEM. SEM analyses were performed with cells from at least three different Transwells. (**c**) Live cell imaging of cells grown under perfusion also showed the high differentiation grade of these cells. The surface of live epithelial cells grown under perfusion was stained using wheat-germ-agglutinin (WGA, green), while intracellular staining comprised mitotracker for mitchondria (red) and Höchst as nuclear stain (blue). For CLSM at least 5 different areas each containing >35 cells per condition were captured. (**d**) Significantly higher TEER values were measured under perfusion in NHBE and SAE cells already after 4 and 6 days compared to static cultures. Each time point represents at least 6 values from 3 different Transwells and significant differences between the mean values of static vs. perfused conditions were analyzed using Unpaired Student’s T test.

Under perfused culture conditions normal human bronchial epithelial (NHBE) (Fig. 1a, upper panel, left) cells showed highly developed tight junctions (red, Occludin) and high mucus production (lilac, MUC5B) after only 7 days in ALI. In contrast, on day 7 under static conditions in ALI epithelial cells exhibited a decreased level of differentiation with no mucus production at all (Fig. 1a, left, middle panel, lilac). Lower tight junction expression was analyzed on day 7 under static conditions (Fig. 1a, left, middle panel, red). Also after three weeks in ALI mucus production (lilac, MUC5B) of epithelial cells grown under static conditions was still not comparable to day 7 perfused cells, while tight junctions (red, Occludin) were similar (Fig. 1a, left, lower panel). In all panels, nuclei were stained using Draq5, a far-red fluorescent DNA dye (Fig. 1a, left, blue).

Under perfused conditions we observed higher ciliogenesis compared to static conditions on day 7 in ALI as analysed by scanning electron microscopy (SEM) (Fig. 1b) and live cell imaging (Fig. 1a, right, Fig. 1c, Video 1 and Video 2). SEM illustrated a higher density of cilia on the surface of epithelia grown under perfusion, while under static conditions cilia were analysed only marginally at this time point (Fig. 1b). Similar results were obtained when surface staining live NHBE cells cultured under perfused (Fig. 1a, right, and Video 1a) or static (Fig. 1a, right, and Video 1b) conditions using fluorescently labelled wheat germ agglutinin (WGA), which binds to glycoproteins of the cell membrane. Cilia movement of live cells grown under ALI and perfusion and stained with WGA (red) and mitotracker (green) after addition of latex beads is illustrated in Video 2. Figure 1c depicts the high cilia development (green) of mitotracker-stained living cells (red) grown under perfusion. Nuclei stained with Höchst are illustrated in blue (Fig. 1c). Similar results were obtained using small airway epithelial (SAE) cells grown under perfused or static conditions. The integrity of SAE cells grown under perfusion is illustrated in Supplementary Fig. S1a and b.

We show here that development and differentiation can be significantly accelerated for lung epithelial cells independent of their origin when they are cultured and grown under perfusion.

### Epithelial differentiation in ALI and under perfusion illustrates a continuous increase in barrier function

A continuous increase in trans-epithelial electrical resistance (TEER), which is a functional parameter to monitor the quality of epithelial cells, such as membrane integrity and various stages of growth and differentiation, was measured over time using cells grown under perfusion or static conditions (Fig. 1d). Measurements on NHBE and SAE cells were taken on several days after ALI (Fig. 1d). TEER values obtained were corrected for the resistance and surface area of Transwell filters. TEER values of NHBE and SAE cells showed a constant increase over time in particular for cells grown under perfusion, and values of NHBE cells increased faster compared to SAE cells (Fig. 1d). From day 21 on TEER values in perfusion and static conditions were comparable and remained stable in long-term cultures (day 21: 1322.7 ± 89.5 Ω*cm^2^ in perfusion, 1263.5 ± 76.7 Ω*cm^2^ under static conditions). These experiments implied the integrity of the epithelial membrane during long-term culture under ALI and perfusion and illustrate the superiority of this system regarding accelerated epithelial cell development.

### Primary allogeneic antigen-presenting cells (DCs, macrophages) are not activated after addition to highly differentiated respiratory epithelial cells

**Figure 2:**
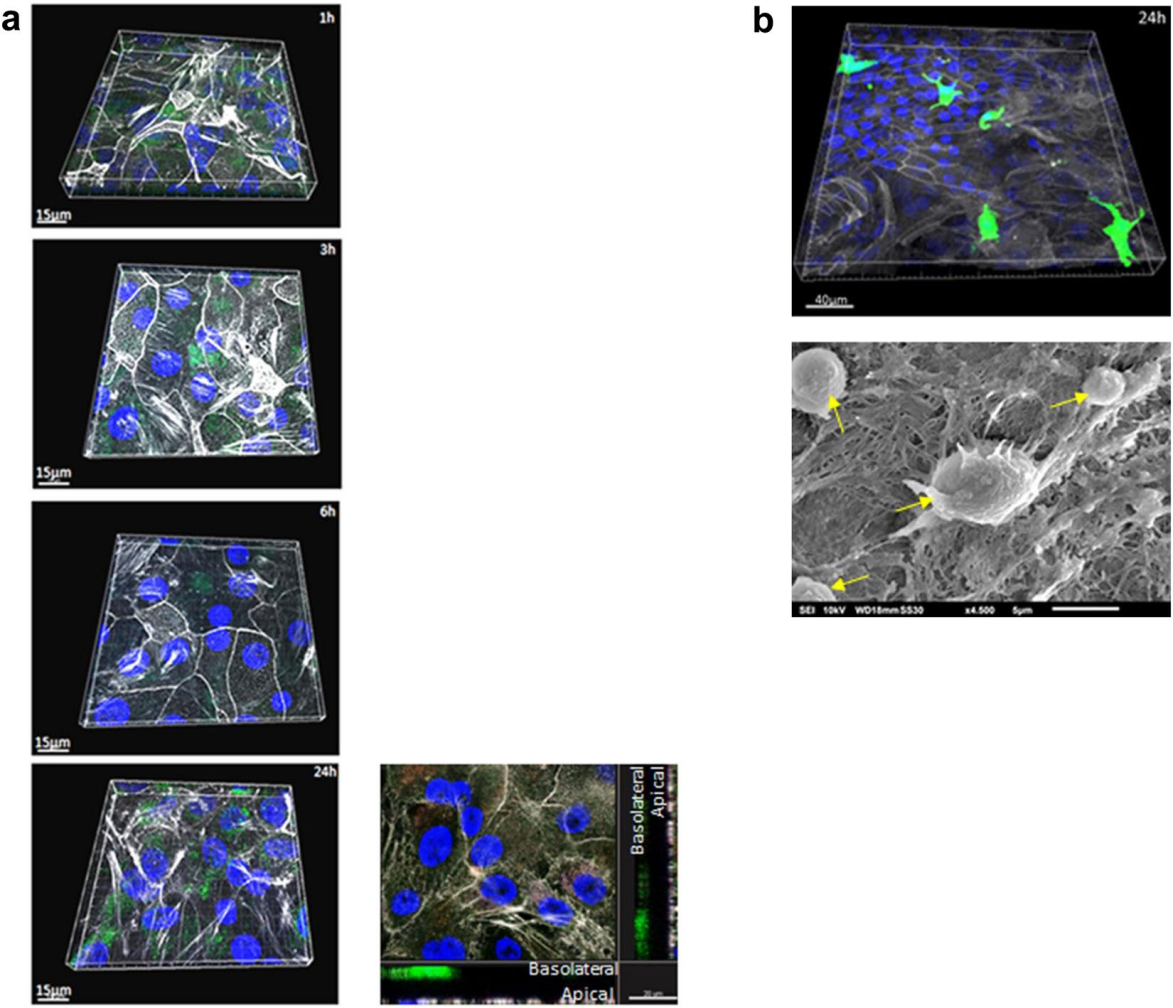
Z-stack series of basolaterally added DCs and apically applied macrophages to analyse the distribution in the co-culture system without any stimulus. The distribution of monocyte-derived DCs (**a**) or macrophages (**b**) (2.5 × 10^5^ cells/Transwell) was analysed over time via CLSM. Tight junctions of epithelial cells were stained using actin (white), nuclei using Höchst (blue) and immune cells using CFSE (2.5 µM) prior to additon to epithelia. (**a**) DCs did not migrate from the basolateral side of the Transwell to the apical layers over a time course of 24 h (1h-3h-6h-24h) and as analysed by x-y projections with respective side views – an example is given at the lower right. (**b**) Apically added macrophages (green) did not show any signs of activation, e.g. clustering on the SAE layer (actin, white; nucleus, blue) even after 24 h as analysed by CLSM (upper) and SEM. Yellow arrows indicate macrophages on the apical side of the epithelium. For CLSM at least 3 different areas each containing ~25 cells per condition were captured.

In a next step, we placed the differentiated epithelial cells grown in fast-track under perfusion into static conditions and basolaterally applied DCs to NHBE or macrophages apically to SAE cells. To exclude unspecific stimulation of the allogeneic immune cells within the system, migration characteristics of antigen-presenting cells (APCs) was monitored in unstimulated co-cultures. Immune cells were fluorescently labelled using Carboxyfluorescein succinimidyl ester (CFSE), a cell-permeable dye covalently coupling to intracellular molecules via the succinimidyl group^21^. CFSE-labelled monocyte-derived DCs (2.5 × 10^5^ cells/Transwell) were adhered to the basolateral side of the epithelium for 1 h and unspecific DC migration without challenge to the apical surface was monitored over time (1 h-3 h-6 h-24 h) using confocal laser scanning microscopy (CLSM) and 3D reconstructions (Fig. 2a). Although about 10–15% of DCs without challenge were attracted to the lower layer of the epithelium through the Transwell filter, x-y projections with respective side views (Fig. 2a, 24 h) revealed that they did not non-specifically migrate to the apical layer of the epithelium at any of the time points measured, as reported after use of cell lines for co-culture experiments^22–24^.

Monocyte-derived and CFSE-labelled macrophages (2.5 × 10^5^ cells/Transwell) were added under static conditions to the apical layer of SAE cells, which - prior to that - were differentiated in ALI and under perfusion. Clustering of macrophages at the apical small airway epithelium due to unspecific activation was excluded in unstimulated co-cultures at the 24 h time point, as analysed by CLSM (Fig. 2b, upper panel) and SEM (Fig. 2b, lower panel). These experiments revealed that APCs added to fully differentiated NHBE or SAE cells did not show any signs of activation under control conditions.

### Myeloid DCs sense fungal pathogens through the epithelial barrier network

**Figure 3:**
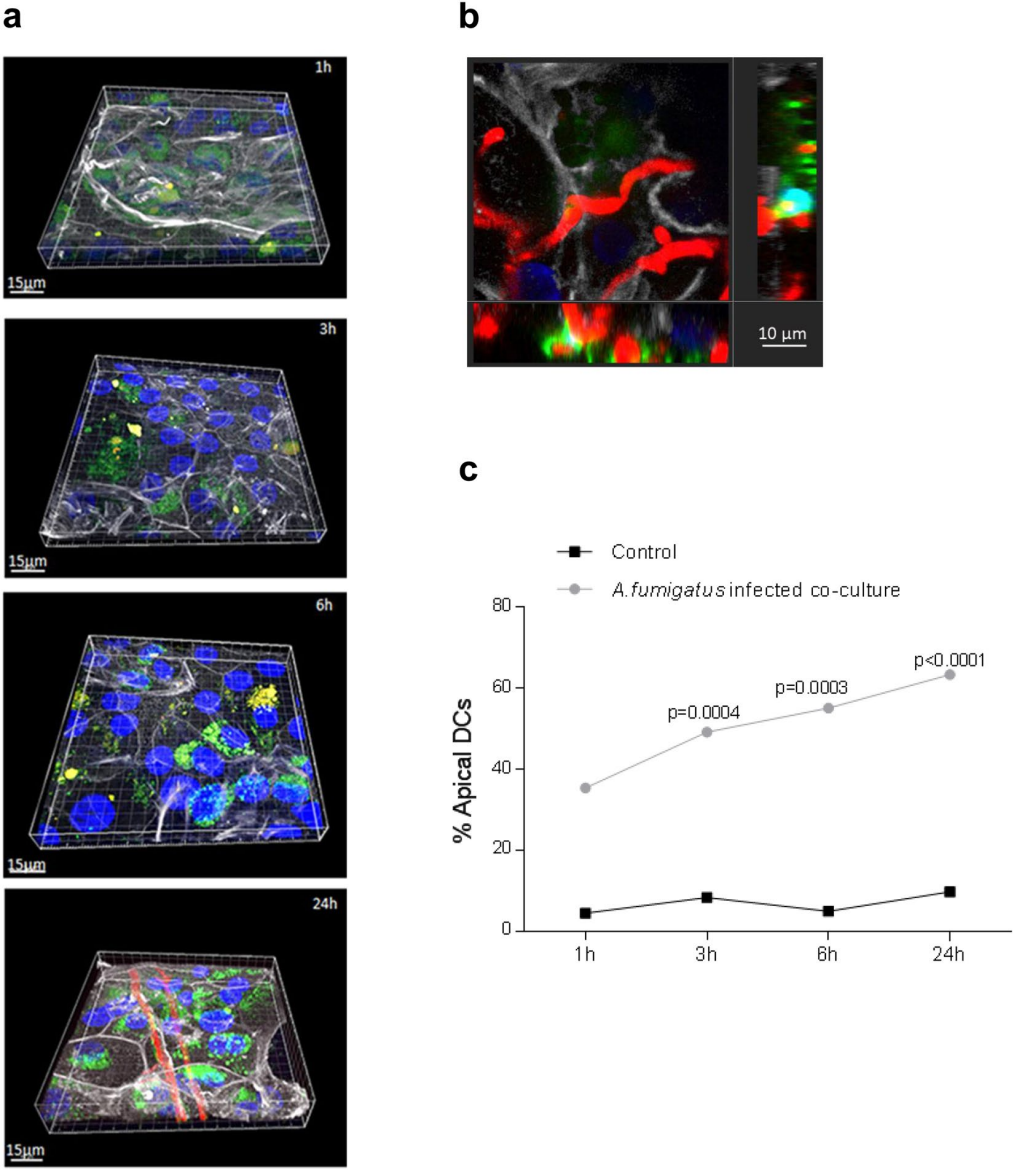
Z-stack series of DC migration through epithelia upon fungal infection. (**a**) Upon fungal infection, DCs (green) sense swollen conidia, germlings (1 h to 6 h, 1^st^ to 3^rd^ panel) and hyphae (24 h) (red) at the apical side of differentiated epithelia (white) and transmigrate over time to the apical side and upon destruction of the epithelium. Actin is illustrated in white, conidia/hyphae in red, nuclei in blue and DCs in green. (**b**) The image shows an x-y projection with the respective side views of DCs (green) interacting with hyphal filaments (red) at the surface of destroyed epithelial cells (white) at the 24 h time point. Bars, 10 µm. CLSM was performed capturing at least 3 different areas each containing more >25 cells per condition. (**c**) Percentages of apically visible DCs over time (1 h to 24 h) in infected DC/NHBE co-cultures are depicted. Significantly more DCs were found at 3 h, 6 h and 24 h at the apical layer compared to background percentages of control cells. Control cells remained at the basolateral side and no increase in DC numbers at the apical layer was detected. Numbers of DCs counted were ~125. Significant differences between infected and control co-cultures were calculated using an unpaired Student’s T test and the Graph Pad Prism software.

We now focused on the functionality of CFSE-labelled DCs in the NHBE model as the most potent APCs and only cells capable of activating naïve T cells and priming antifungal adaptive immune responses^7, 8, 15, 23, 24^. We applied dsRed-*A. fumigatus* conidia (0.5 × 10^6^; MOI 0.5) to the apical side of the epithelium and monitored co-localization of dendrites or DCs over time using CLSM (Fig. 3). At various time points after infection (1 h, 3 h, 6 h and 24 h), cells were fixed and additionally stained for actin (white) and nuclei (blue). Images were obtained in Z-stacks and 3D reconstructions were performed using the IMARIS 8.2.0 software. In contrast to non-infected controls, where only background levels of DCs were found in apical layers over time (Figs 2a and 3c), in infected co-cultures first dendrites co-localized with swollen dsRed-conidia already at the 1 h time point (Fig. 3a, first panel). At 3 h and 6 h (Fig. 3a, second and third panel), more co-localization (yellow) of fungi (red) with DCs (green) was analysed within the epithelial/immune cell co-cultures. At a later time point (24 h), destruction of the epithelium was clearly visible (Fig. 3a, forth panel) due to hyphal overgrowth of the Transwell, which caused migration and aggregation of DCs alongside the hyphae – co-localization (yellow) of CFSE-labelled DCs (green) alongside hyphae (red) is also illustrated in an x-y projection with the respective side view (Fig. 3b). In Fig. 3c percentages of DC migration upon fungal infection are illustrated. At 1 h post infection more DCs were detected in the apical layer compared to control DCs, but differences between conditions were not significant. DC signals after 3 h, 6 h and 24 h of *A. fumigatus* infection illustrated a significant difference compared to control cells and cell numbers increased at the apical side over time (Fig. 3c). We here showed that upon *A. fumigatus* infection, high percentages of DCs migrate to the apical layers over time. In contrast low percentages of DCs in non-infected co-cultures and slightly higher DC percentages at early time points after infection were found associated with the basolateral layers.

### *A. fumigatus* mediates increased expression of IL-8 and RANTES in differentiated NHBE cells

**Figure 4:**
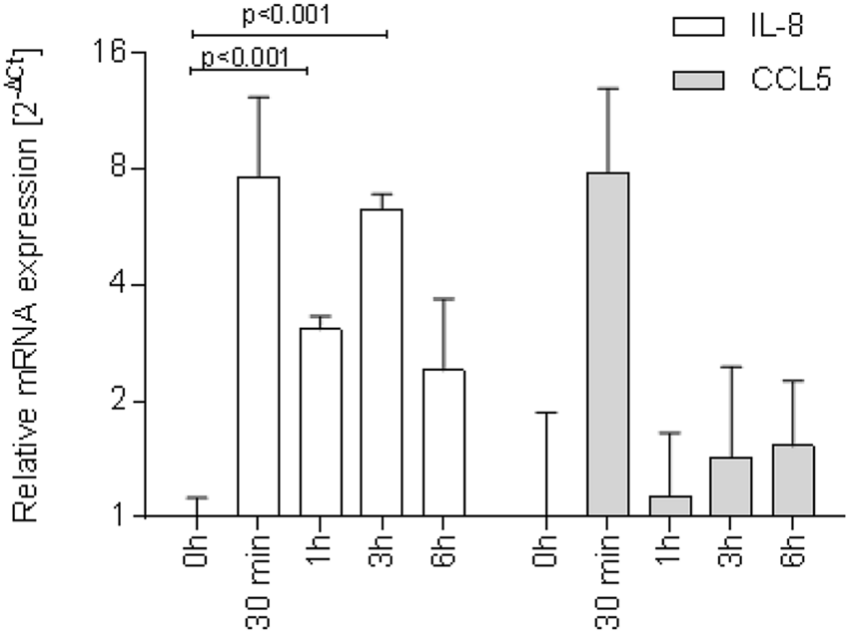
Immediate inductions of RANTES and IL-8 mRNA expression in NHBE cells upon fungal infection. Fungal infection of differentiated NHBE cells (MOI 0.5) mediates a significant, immediate increase in IL-8 (white bars) and CCL5 (RANTES) (grey bars) mRNA expression levels after 30 min. IL-8 expression remains elevated up to 6 h post infection, indicating the release of the pro-inflammatory cytokine over time to induce immune cell migration to sites of infection. In contrast, RANTES levels dropped to nearly background expression at 0 h (grey bars). Cytokine expression was measured from three independent Transwells and qRT-PCR was performed in duplicates. Significant differences between infected and control co-cultures were calculated using an unpaired Student’s T test and the Graph Pad Prism software.

To analyse whether sensing of *A. fumigatus* swollen conidia (3 h), germlings (6 h) or hyphae (24 h) by DCs and their subsequent activation is due to production of cytokines by the epithelium or by higher expression of pathogen-associated molecular patterns (PAMPs) expressed by hyphae, we next characterized the expression levels of pro-inflammatory and chemoattractant mediators (IL-8, CCL5 [RANTES]) in highly differentiated NHBE cells and mediated by the various morphotypes of *A. fumigatus* (Supplementary Fig. S2). IL-8 as well as RANTES levels were increased in *A.fumigatus-*exposed NHBE cells already after 30 min. IL-8 and RANTES expressions were highest at this short-term time point, and IL-8 levels remained elevated compared to non-infected control cells also at 24 h after infection (Fig. 4), while CCL5 mRNA expression levels dropped to background levels. The immediate induction of IL-8 and RANTES in NHBE cells led us to the conclusion that at early time points after infection DCs are attracted by cytokines released from epithelial cells, while at later time points PAMPs may also play a role regarding DC attraction.

### *A.fumigatus*-exposed DCs display a mature phenotype

**Figure 5:**
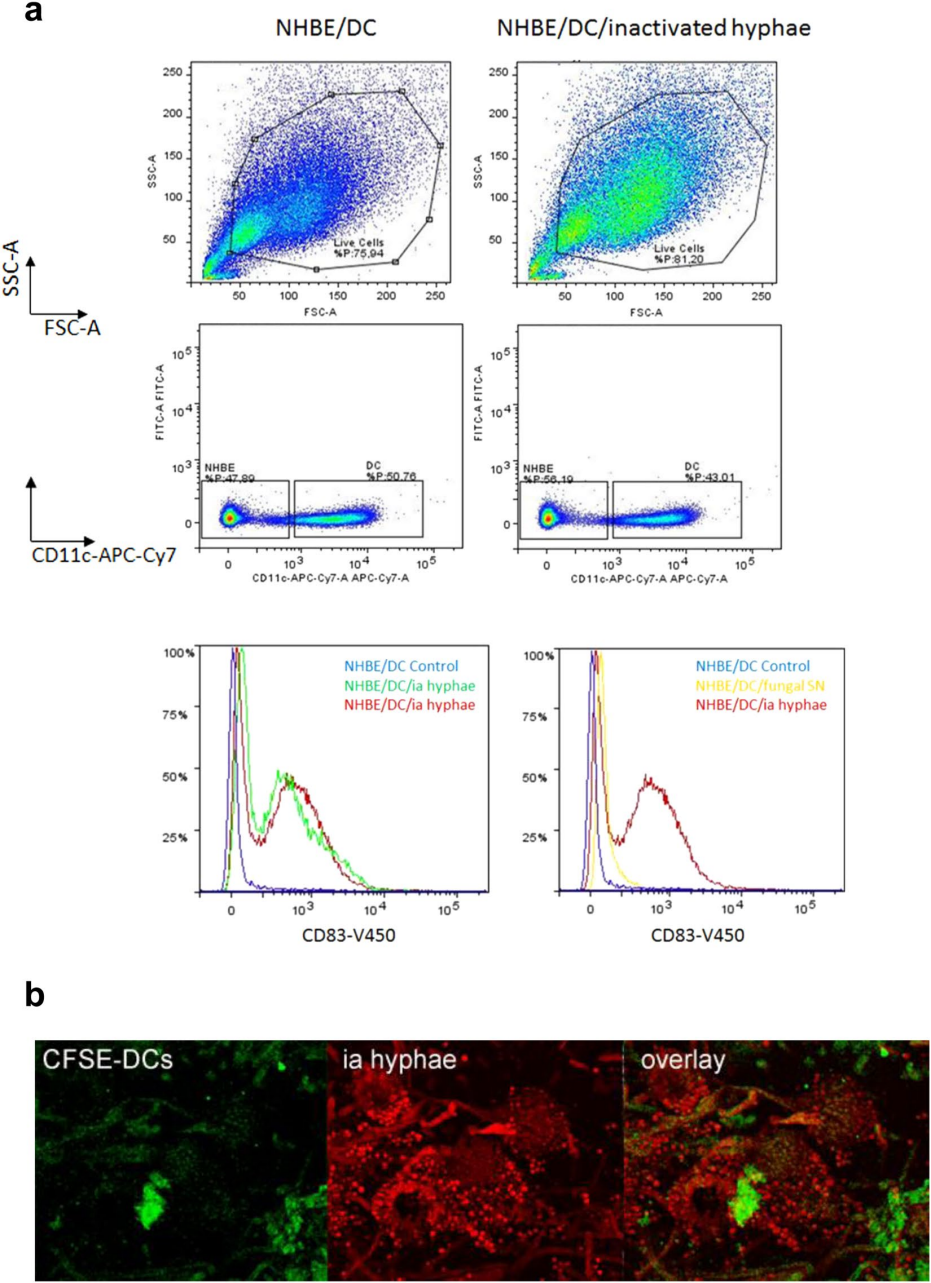
DC maturation and activation upon fungal exposure of the epithelia. (**a**) DC maturation is induced by inactivated (ia) hyphae. Gating of non-infected (upper, left) and *A. fumigatus*-infected (upper, right) NHBE/DC co-cultures revealed that under both conditions about 80% were alive of which ~50% showed a high expression of the characteristic DC marker CD11c, while the other half did not express this marker, thereby characterizing these CD11c-negative cells as NHBE cells (middle panel). The CD11c^high^ population of non-infected and infected co-cultures was further characterized for the DC maturation marker CD83. Only upon apical application of inactivated *A. fumigatus* hyphae a significant up-regulation of CD83 was detectable as shown on cells harvested from two independent Transwells (lower panel, left, red and green), while control cells did not mature (lower panel, left, blue). Also the application of fungal SNs to the apical surface of the epithelium (lower panel, right, yellow) did not cause DC maturation as high as inactivated *A. fumigatus* hyphae (lower panel, right, red). Compared to uninfected control cells (lower panel, right, blue), only a slight shift to the right was observable respecting CD83 upon treatment of the epithelium with fungal SNs (lower panel, right, yellow). FACS analyses were repeated thrice. (**b**) CLSM analyses revealed apical DC attachment to ia hyphae. To see if PAMPs are sufficient for DC attraction, apical epithelial layers were exposed to ia hyphae (red) for 48 h. CFSE-DCs (green) showed a co-localization with hyphae (overlay). CLSM analyses using ia hyphae and counting at least 25 cells were performed twice.

Lastly, we analysed whether DCs exhibit a mature phenotype upon fungal exposure within the co-culture system. For this, UV-inactivated hyphae or *A. fumigatus* supernatants (SNs) were applied to the apical side of the epithelial barrier and DCs were exposed to these stimuli for 48 h. UV inactivation of *A. fumigatus* or application of SNs allowed the long-term treatment within the system without fungal overgrowth of the Transwell co-culture and to see, whether PAMPs on hyphae are sufficient for DC activation. All cells were harvested from the Transwell and in addition migrated DCs from the lower chamber of the Transwell were collected. This lower chamber contained the CCR7 ligands CCL19 and CCL21 [100 ng/ml] to mimic migration of fully mature DCs (mDCs) to the lymph nodes after pathogenic stimulation^25, 26^. All cells were pooled to analyse the levels of matured DCs within the system. DCs were specified using CD11c as a characteristic marker not expressed on NHBE cells and CD83 as a specific marker of dendritic cell maturation (Fig. 5a). Approximately 80% of the cells were alive under control (Fig. 5a, upper left panel) and infected conditions (Fig. 5a, upper right panel). DCs and epithelial cells were differentiated by their CD11c^+^ expression and CD11c^high^ cells, representing DCs (Fig. 5a, middle panel), were further analysed for their CD83 expression (Fig. 5a, lower panel). These analyses revealed that upon apical stimulation of NHBE/DC co-cultures using inactivated (ia) hyphae, CD83 was highly expressed on DCs. This higher expression of CD83 was not found under un-infected control conditions. Figure 5a (lower left panel) illustrates an overlay of CD83 expression on DCs from two independent, *A.fumigatus*-infected Transwells (NHBE/DC/ia hyphae, red and green) compared to a non-infected Transwell control (NHBE/DC Control, blue). Further, we applied fungal supernatants to the apical side of the Transwell and compared DC maturation of these co-cultures with maturation mediated by ia hyphae. In contrast to fungal supernatants (Fig. 5a, lower right panel, NHBE/DC/fungal SN, yellow) and non-infected control cells (Fig. 5a, lower right panel, NHBE/DC Control, blue), in co-cultures with NHBE cells DCs exposed to inactivated hyphae showed an up-regulation of the characteristic DC marker CD83 (Fig. 5a, lower right panel, NHBE/DC/ia hyphae, red). In addition CLSM analyses illustrated attachment, grabbing (Fig. 5b, upper panel) and aggregation (Supplementary Fig. S3) of CFSE-DCs (green) and inactivated hyphae (red) on the epithelial cell layer after 48 h (Fig. 5b). Inactivation of hyphae was further confirmed by live/dead staining (Supplementary Fig. S3).

These analyses revealed that in contrast to (inactivated) hyphae, fungal supernatants at this time point and after single addition were not capable of causing functional maturation of DCs within the epithelial/immune cell co-culture system.

### Macrophages sense fungal pathogens in NHBE dual cultures

**Figure 6:**
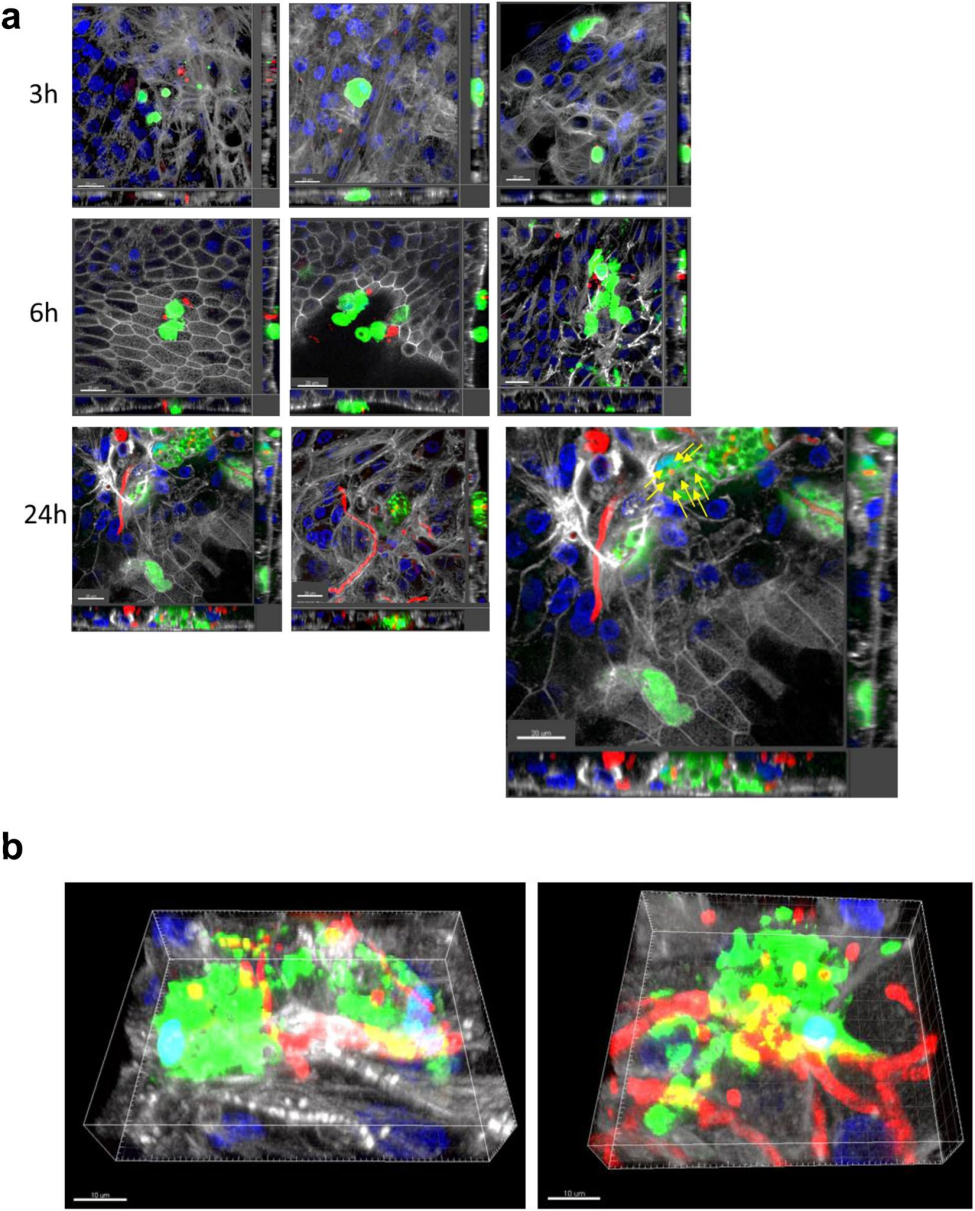
Z-stack image series to analyse the phagocytic activity of apically applied monocyte-derived macrophages upon infection with A. fumigatus in co-culture with SAE cells. Co-cultures of SAE cells (white) grown under perfusion were transferred to static conditions and CFSE-labelled monocyte-derived macrophages (green) were apically applied prior to infection with A. fumigatus conidia (0.5 × 10^6^; MOI 0.5) for (**a**) 3 h, 6 h and (**a**) and (**b**) 24 h. (**a**) At 3 h (upper panel), single apically applied macrophages start migrating towards swollen conidia with single conidia already internalized into macrophages (arrow). At 6 h (middle panel) macrophage cluster around swollen/germinating conidia, while at 24 h (lower panel) many conidia were taken up by macrophages (arrows), which were also aggregating around hyphae on the SAE epithelium (**b**) x-y projections with respective side views are depicted in (**a**), while (**b**) shows 3D reconstructions at the 24 time point.

We further checked the functionality of apically added macrophages (CFSE-labeled; green) in a comparable model of fast-track generated small airway epithelial cells to act against fungal pathogens (dsRed *A. fumigatus*, red). We also applied fungal conidia (0.5 × 10^6^; MOI 0.5) to the apical side and monitored interactions of macrophages and fungi using CLSM over time (Fig. 6a). At various time points after infection (3 h, 6 h and 24 h), cells were fixed and stained for actin (white) and nuclei (blue), images were obtained in Z-stacks and 3D reconstructions using IMARIS 8.2.0 software were performed (Fig. 6b). A high number of macrophages formed aggregates with swollen or germinating fungal conidia (3 h, 6 h) as well as hyphae (24 h) while in uninfected co-cultures macrophages were evenly distributed on the epithelium and did not form clusters (Fig. 2b). Furthermore, percentages of internalized swollen or germinated fungal conidia by macrophages were calculated. After 3 h, 13.3% of conidia were internalized and the numbers increased to 28.2% at 6 h. At 24 h, calculation of internalized pathogen percentages was not possible anymore, since the fungi already formed hyphae. Nevertheless multiple phagocytized conidia were detectable within macrophages at this time point (Fig. 6a, magnification with yellow arrows pointing to multiple internalized conidia within one macrophage). Macrophages in uninfected co-cultures were evenly distributed as single cells throughout the apical surface of differentiated epithelia (Fig. 2b). Macrophages efficiently sensed pathogens at the apical surface of the epithelium, thereby making the fast track co-culture of SAE/immune cells a convenient model to study infections of the lower respiratory tract in a more relevant *in vitro* system.

## Discussion

Here, we describe the set-up of a novel fast-track *in vitro* approach of human primary respiratory tract cells grown in ALI and under perfusion and containing myeloid DCs or macrophages to study airborne infections, allergies or toxic effects of inhaled particles of the upper and lower respiratory tract. During recent years, a large number of 3D cell culture models of the upper or lower respiratory tract composed of two to four different cell types have been published^23, 24, 27–30^. Most of these mixed models were composed of cell lines such as A549, HMC-1, LLC, THP-1, often submerged but not ALI, and these conditions also exerted an impact on the experimental outcome^31–33^. Cell lines often do not represent the features and functions of primary cells with respect to morphologic, biochemical and genetic characteristics of the original tissue. The benefit of using primary cells in the system compared to established cell lines is that the latter exhibit higher similarity to the original cells/tissues. This is shown by the heterogeneity of the normal human bronchial epithelial layer comprised of goblet, basal, ciliated and non-ciliated cells. Furthermore, along the upper and lower respiratory tracts a contiguous DC network exists^34^, that differs in its function between these two tracts due to the commonly sterile nature of the bronchioles and alveoli in the lower respiratory tract^35, 36^. Upper respiratory tract DCs represent myeloid DCs, expressing high levels of CD11c, MHC II and fluctuating CD11b levels^35^.

Monocytes rapidly infiltrate lung tissues after pathogen exposure and differentiate to inflammatory DCs, thereby contributing to the DC pool^37, 38^. To characterize the role of antigen-presenting cells within the lung epithelium in more detail and within an accelerated procedure, we co-cultured monocyte-derived DCs with upper respiratory tract or macrophages with small airway epithelia. These more likely reflect their myeloid counterparts. Improvement of the modified *in vitro* system not only occurred on the cellular level but also with respect to culture conditions – when comparing development and differentiation of normal human bronchial and small airway epithelial cells grown in ALI under static or perfused conditions, we demonstrated here that epithelial cells grown under perfusion exhibited a significantly accelerated ciliogenesis and mucus production, since already at day 7 post ALI culture all these features were well established in contrast to 21 days under static conditions. Static conditions on day 21 in ALI also exhibited less mucus production compared to perfused conditions on day 7 in ALI. The use of perfused dynamic conditions allows re-circulation of conditioned medium, thereby providing a constant supply of nutrients and enrichment of growth factors, which – as shown here - results in faster development and higher differentiation of the lung epithelia, independent of their origin.

A major function of DCs is the induction of an efficient adaptive immune response in lymph nodes (LNs)^39–42^. DCs also have to sense particles or soluble antigens/allergens persisting at epithelial surfaces and they are attracted to these sites due to chemotactic mediators, i.e. IL-8 or CCL5 [RANTES], secreted by epithelial cells, which we also detected in our system, or due to direct cell-cell communication^43^. To test the communication of the major antigen-presenting cells, i.e. DCs and macrophages, in our system we applied *A.fumigatus* conidia or inactivated hyphae to the apical surface of NHBE or SAE cells differentiated in fast-track under perfusion and observed the antigen-capture and phagocytosis or after longer time points also migration of DCs through the damaged epithelia. These experiments revealed that the DCs first sensed the fungal particles via their dendrites through the epithelium, but as soon as the fungi started to swell, germinate and form hyphae at the surface of the epithelium, an increased number of DCs was found also at apical sites of infection. If using a tumor cell line e.g. Lewis lung carcinoma (LLC) cells as an airway epithelial cell model, cell transmigration through the epithelium to the apical layer was illustrated to be induced without any additional stimulus, since tumor cells were shown to secrete monocyte-chemoattractant protein (MCP)^23, 44–46^. In our system using NHBE or SAE cells in combination with primary DCs or macrophages, we did not detect any transmigration or activation of the immune cells in the absence of a stimulus, while upon pathogenic stimulation the cells were activated as analysed by their migration properties, maturation, internalization of swollen conidia or interactions with hyphae. These results emphasize the importance of the right choice of cells when designing such complex multi-cellular systems to avoid *in vitro* artefacts due to unspecific effects auto-produced by the system.

In summary, we were able to demonstrate that in our *in vitro* 3D upper and lower respiratory tract system consisting of highly differentiated epithelial cells grown in ALI and under perfusion, primary DCs or macrophages are fully functional and fulfil their tasks of sensing, phagocytizing and sampling antigens present at the apical surface of epithelial cells. Of course, it has to be noted, that currently the lower respiratory tract model does not include the alveolar surface, but only small airway epithelial cells.

The development of innovative, animal-free approaches in a scientific context is of high importance due to ethical issues and physiological relevance. An *in vitro* upper and lower respiratory tract model from normal human bronchial or small airway epithelial cells grown in ALI and under perfusion was further refined due to insertion of relevant primary antigen-presenting cells, such as dendritic cells and macrophages. The use of primary cells within the system avoids unspecific effects seen in models using cell lines for such models, such as secretion of chemoattractants seen by tumor cell lines. Additionally, primary differentiated cells illustrate a surface marker expression as found in the body and not one characteristic for a mixed population, which is the case for THP-1-generated macrophages or DCs. The growth of the epithelial cells in ALI and under perfusion demonstrated an improved and accelerated ciliogenesis and production of an intact muco-ciliary layer as well as an improved barrier function compared to cells grown under static conditions, thereby allowing more streamlined and faster further processing. APCs were fully functional in terms of sensing pathogens, phagocytosis and migratory characteristics upon infection with airborne *A. fumigatus*. Thereby, fast-track development of highly differentiated lung epithelial cells due to applying perfusion significantly facilitates further applications with respect to co-cultures with immune cells for studying airborne challenges.

## Materials and Methods

### NHBE or SAE cell culture

Primary normal human bronchial epithelial (NHBE) or small airway (SAE) cells were obtained from Lonza. The cells were cultured in a T75 flask for 2–4 days until they reached 80% confluence. The cells were trypsinised and seeded onto collagen (Corning collagen I, Rat tail)-coated 0.33 cm^2^ porous (0.4 µm) polyester membrane inserts with a seeding density of 1 × 10^5^ cells per Transwell (Costar, Corning). The cells were grown to near confluence in submerged culture for 2–3 days in specific epithelial cell growth medium according to the manufacturer´s instructions. Cultures were maintained in a humidified atmosphere with 5% CO_2_ at 37 °C and then transferred to ALI culture. The epithelium was fed with B-ALI or S-ALI differentiation medium (Lonza). The number of days in development was designated relative to initiation of ALI culture, corresponding to day 0. Cells were used between day 50 to day 70 in ALI and under perfusion.

### Dynamic perfused cell culture

A commercially available ALI perfusion chamber bioreactor (Quasi-Vivo, QV600, Kirkstall, United Kingdom) was used to keep the cells under perfusion. The main feature of this system is the ability to apply various flow rates dependent on the cell type and providing constant nutrient turnover to cells without imposing high shear stress or turbulent flow^47^. After pre-culturing NHBE or SAE cells on Transwells under submerged conditions as described above, inserts were transferred into the QV600 chamber (ALI) (Kirkstall). Quasi-Vivo chambers and a mixing chamber (ALI medium reservoir) were then connected in series to a peristaltic pump (Ismatec). The medium was pumped through the chambers at a rate of 150 µl/min and simultaneously maintaining an air-liquid interphase (ALI). The medium in the reservoir bottle was re-fed every week with specific medium to maintain a final volume of 25 ml.

### Monocyte-derived macrophage and dendritic cell (DC) culture

Monocytes were isolated from the blood of normal healthy donors by using CD14 BD IMAG Beads (Becton-Dickinson), according the manufacturer’s instructions. Monocytes were washed and cultivated in RPMI 1640/10% FCS/2 mM l-glutamine (RPMI_c_) containing 1500 U/ml IL-4 and 1600 U/ml GM-CSF at a density of 1 × 10^6^ cells/ml medium in six-well plates (Costar) to generate monocyte-derived DCs. IL-4 (1000 U/ml) and GM-CSF (1600 U/ml) were added to the medium after 2 days in culture. Day 5 DCs were harvested for generating dual transwell cultures^48^. Monocytes were also differentiated into macrophages for 6 days in RPMI 1640/5% FCS/ 1% l-glutamine (RPMI_c_) containing 50ng/ml GM-CSF at a density of 1 × 10^7^ cells in T-75 flasks. Day 6 macrophages were harvested for generating dual transwell cultures.

### TEER measurement

TEER values were measured using EVOM voltohmmeter with STX-2 chopstick electrodes (World Precision Instruments, Stevenage, UK). Measurements on cells in ALI culture were taken immediately before the medium was exchanged. For measurements, 0.5 ml and 1.0 ml of medium were added to the apical and basolateral chambers, respectively. Cells were allowed to equilibrate before TEER was measured. TEER values reported were corrected for the resistance and surface area of the Transwell filters.

### Generation of DC-NHBE dual Transwell cultures

For dual co-culture experiments, NHBE cells were grown on collagen-coated Transwell inserts under perfused conditions as described and subsequently 2.5 × 10^5^ DCs/Transwell were allowed to adhere to the basolateral surface of the insert for one hour. Prior to adherence, DCs were stained using 2.5 µM CFSE for detection by confocal microscopic analyses. After 1hr adherence, the inserts were reverted back to their original position and the co-cultures were infected with 0.5 × 10^6^ dsRed *A. fumigatus* conidia to mimic fungal infection of the upper respiratory tract. 20 µl of the conidial suspension were added to the apical side of the co-cultures and incubated at 37 °C for various time points (30 min to 24 h dependent on the experimental set up). Inocula were removed by gentle suction and fixed with 4% paraformaldehyde solution for 10 minutes. After fixation the cells were washed three times with D-PBS solution, permeabilised for 15 minutes (eBioscience) followed by blocking with 1% BSA. Actin localized to tight junctions was identified by using phalloidin-650 (1:20; Cell Signaling Technology) and nuclei were stained using Hoechst 33342 (Cell Signaling Technology). Images were captured using a Leica SP5 confocal laser scanning microscope and analysed using the LAS AF Lite software (Leica).

### Generation of Macrophage-SAE dual Transwell cultures

For dual co-culture experiments, SAE cells were grown on collagen-coated Transwell inserts under perfused conditions as described and subsequently 2.5 × 10^5^ macrophages/well were allowed to adhere to the apical surface of the insert for two hours. Prior to adherence, macrophages were stained using 2.5 µM CFSE for detection by confocal microscopic analyses. After 2 h adherence, the medium from the apical surface was gently removed and the co-cultures were infected with 0.5 × 10^6^ dsRed *A. fumigatus* conidia to mimic fungal infection of the lower respiratory tract. 20 µl of the conidial suspension were added to the apical side of the co-cultures and incubated at 37 °C for various time points (1 h to 24 h dependent on the experimental set up). Inocula were removed by gentle suction and fixed with 4% paraformaldehyde solution for 10 minutes. After fixation the cells were washed three times with D-PBS solution, permeabilised for 15 minutes (eBioscience) followed by blocking with 1% BSA. Actin localized to tight junctions was identified by using phalloidin-650 (1:20; Cell Signaling Technology) and nuclei were stained using Hoechst 33342 (Cell Signaling Technology). Images were captured using a Leica SP5 confocal laser scanning microscope and analysed using the LAS AF Lite or Imaris software.

### Fungal strains

Wild-type *A. fumigatus* ATCC46645 (see Supplementary Fig. S2) was used for scanning electron microscopy (SEM) analysis, and dsRed *A. fumigatus* ^49^ (see Supplementary Fig. S4) were used for CLSM. Conidia were harvested from *Aspergillus* complete medium agar plates after 7 days at 37 °C. *Aspergillus* complete medium is composed of 20 g glucose, 2 g peptone, 1 g tryptone, 1 g yeast extract, 20 ml 50 × salt solution (50 × salt solution is [per liter] 26 g KCl, 26 g MgSO_4_·7 H_2_O, 76 g KH_2_PO_4_), 1 ml trace element stock solution (trace element stock solution is [per liter] 0.04 g Na_2_B_4_O_7_·10H_2_O, 0.4 g CuSO_4_·5H_2_O, 0.741 g MnSO_4_·H_2_O, 8 g ZnSO_4_·7H_2_O, 0.8 g Na_2_MoO_4_·2H_2_O), and 12.5 g agar per litre (pH 6.5)^50^. The conidial suspension was harvested by flooding each colony with 2 ml spore buffer solution (0.9% NaCl and 0.1% Tween 20). The conidial suspension was filtered through a PARTEC filter and centrifuged at 18360 RCF at 4 °C for 5 minutes. The pellet was resuspended in RPMI 1640 medium and the conidia were counted with a hemocytometer. For SEM and CLSM 0.25 × 10^6^ conidia were used.

For inactivated hyphae, 2 × 10^6^ conidia were inoculated in 20 ml medium in a petri dish and incubated overnight at 37 °C. The supernatant was discarded, the petri dish containing hyphae was washed three times before inactivation by ultraviolet (UV) light for 60 min. The hyphae matt were transferred to the apical side of the NHBE/DC co-cultures.

### *Aspergillus* infection and microscopic analyses

For CLSM analysis, 0.5 × 10^6^ ds Red *A. fumigatus* conidia were applied to the apical surface and at several time points post infection as indicated in the Figure legends or Results, inocula were removed by gentle suction and the well was fixed using 4% paraformaldehyde solution for 10 minutes. After fixation the cells were treated as described above except staining the slides for mucin using rabbit anti-human MUC5B antibody (1:80; Abcam), followed by donkey anti-rabbit IgG (1:50 Biolegend). Again, actin localized to tight junctions was identified by using phalloidin-650 (1:20; Cell Signaling Technology). Cilia were stained using an anti-tubulin antibody (1:10; BD Pharmingen), and nuclei using Hoechst 33342 (Cell Signaling Technology). Images were captured using a Leica SP5 confocal laser scanning microscope and analysed using the LAS AF Lite or Imaris software. For each condition at least 50 cells were analyzed and used for quantifications.

### Scanning Electron Microscopy (SEM)

For SEM analyses, WT *A.fumigatus* ATCC 46645 (0.5 × 10^6^ conidia) were added to the apical surface and at 5 min, 30 min, 3 h and 5 h post-infection inocula were removed by gentle suction and fixed with 2.5% glutaraldehyde EM Grade R1012 (BioChemika Fluka) in 0.1 M phosphate buffer (pH 7.4) solution overnight. The next day, samples were washed with sterile filtered D-PBS solution and gradually dehydrated using ethanol. Sputter coating was performed with gold and a Jeol6010 in Touch (Jeol Germany) was used for analyses. Samples were examined with a field emission scanning electron microscope (Gemini 982; Zeiss).

### Flow Cytometry

For flow cytometry, inactivated fungal hyphae were applied to the apical side of the epithelium to allow longer incubation times and the DCs sufficient time to get in contact with the fungal pathogens without fungal overgrowth of the cells. The lower chamber of the Transwell was supplemented with the CCR7 ligands CCL19 and CCL21 (100 ng/ml each, Miltenyi Biotech) and cells were harvested after 48 h. We harvested NHBE and dendritic cells from the apical and the basolateral side of the Transwell but also those from the lower chamber, to see if these are mature migrated DCs. Cells were detached using 0.5 M EDTA and run over a cell strainer to remove inactivated hyphae. Cells were then stained using fluorescently labelled CD11c and CD83 mAbs (Biolegend) and fixed for analyses using a BD FACS Verse™ (BD Biosciences).

### Real-time RT-PCR

Cytokines (human IL-8 and CCL5) were analyzed by real-time RT-PCR as described using commercially available gene-specific primer/probe pairs (BioRad). A GAPDH (human)-RT-PCR using commercially available specific primer/probe pairs (BioRad) served as internal controls to quantify the relative gene expression of target genes. The iScript One-Step RT-PCR Kit for Probes (BioRad) was used for target amplification and runs were performed on the CFX96 real-time detection system (BioRad). The cycling conditions were as follows for all analyses: 10 min@50 °C (RT), 3 min@95 °C, 40 cycles: 5 sec@95 °C, 15 sec@60 °C. The results were analyzed using the gene expression software of the cycler (CFX Manager Software, BioRad, ∆∆Ct method).

### Availability of data and materials

Data sharing is not applicable to this article as no datasets were generated or analyzed during the current study.

### Ethics approval and consent to participate

Written informed consent was obtained from all participating blood donors by the Central Institute for Blood Transfusion & Immunological Department, Innsbruck, Austria. The use of anonymized leftover specimens for scientific purposes was approved by the Ethics Committee of the Medical University of Innsbruck. All methods were performed in accordance with the relevant guidelines and regulations.

## Electronic supplementary material

Video 1a

Video 1b

Video 2

Video Legends and Supplementary Figure Legends and Figures 1 to 4
