# Supplementary material for: Fast-track development of an in vitro 3D lung/immune cell model to study Aspergillus infections
Source: Sci Rep. 2017 Sep 14;7:11644. doi: 10.1038/s41598-017-11271-4 (PMC5599647; doi:10.1038/s41598-017-11271-4)

Fast-track development of an *in vitro* 3D lung/immune cell model to study *Aspergillus* infections

Chandorkar P, Posch W, Zaderer V, Blatzer M, Steger M, Ammann CG, Binder U, Hermann M, Hörtnagl P, Lass-Flörl C, Wilflingseder D.

Video Legends

Video 1 Comparison of cilia development and beating between NHBE cells grown in ALI under perfused vs. static conditions. (a) Perfused conditions were superior in ciliogenesis as well as their activities while ***(b)*** under static conditions NHBE cells showed single spots of cilia development and relatively slow beating.

Video 2 Mucociliary clearance and cilia activity analyzed by live cell microscopy. The surface of epithelial cells grown under perfusion was stained using wheat-germ-agglutinin (WGA, red) while intracellularly cells were stained using mitotracker for mitchondria (green).

Supplementary Figure Legends

Supplementary Figure S1a: CLSM and SEM analyses of SAE cells grown under perfusion. The upper and middle panels depict CLSM analyses of SAE cells grown for 7 days under perfusion. Well-developed tight junctions (occludin, white) and cilia formation (lilac, middle panel) are observable as well as high rates of live cells as illustrated by using the Live/Dead Cell Viability Staining (Thermo-Fisher Scientific) (red, upper panel). Nuclei were stained using Draq5 (blue). The lower panel shows SEM of intact SAE epithelia. ***Supplementary Figure S1b: Live cell imaging of SAE cilia movement upon stimulation with beads (snapshot).*** Cilia were stained using WGA (blue), latex beads are depicted in red.

Supplementary Figure S2: Different A. fumigatus (ATCC46645) morphotypes (conidia, swollen conidia, germlings and hyphae) are depicted.

Supplementary Figure S3: CLSM analyses revealed apical DC aggregation around ia hyphae. To see if PAMPs are sufficient for DC attraction, apical epithelial layers were exposed to ia hyphae (red) for 48h. CFSE-DCs (green) were attracted to high numbers by ia hyphae (overlay). In addition a live/dead staining was performed (blue), which illustrated the killing of the applied fungi.

Supplementary Figure S4: dsRed Aspergillus fumigatus hyphae are illustrated by light (left) and fluorescence (right) microscopy.

Supplementary Figure S1a

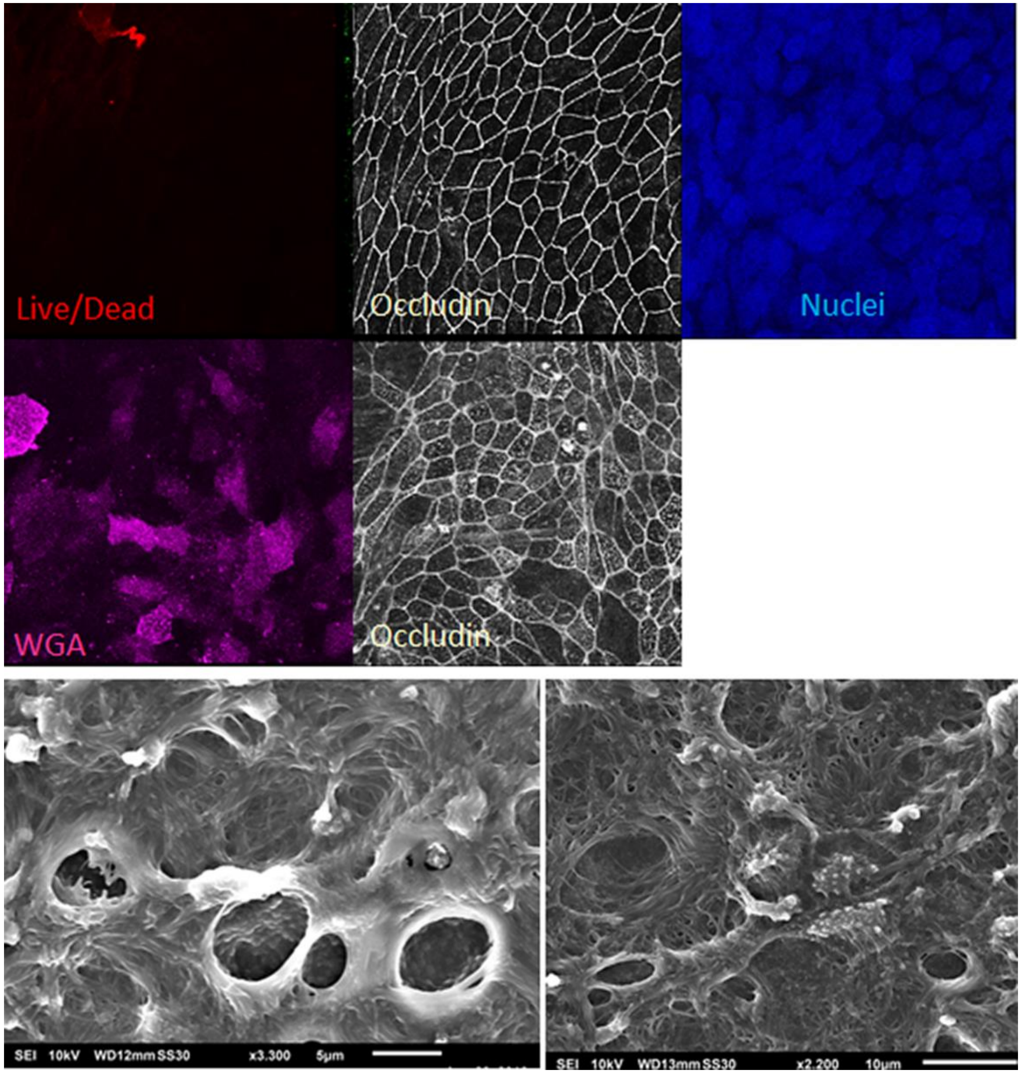

S1b

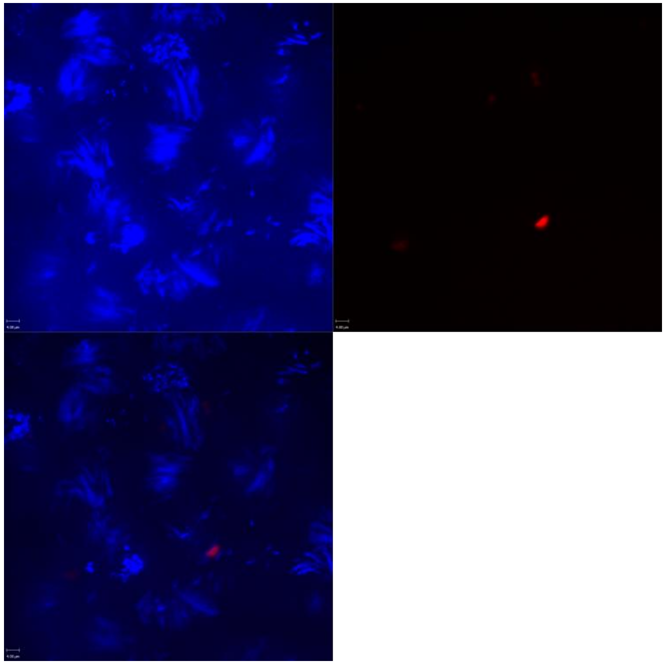

Supplementary Figure S2

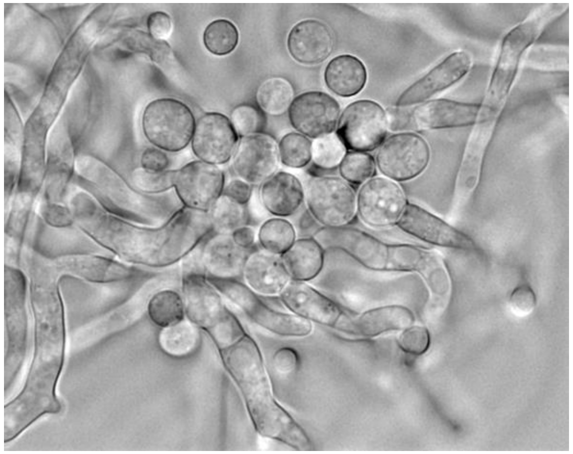

Supplementary Figure S3

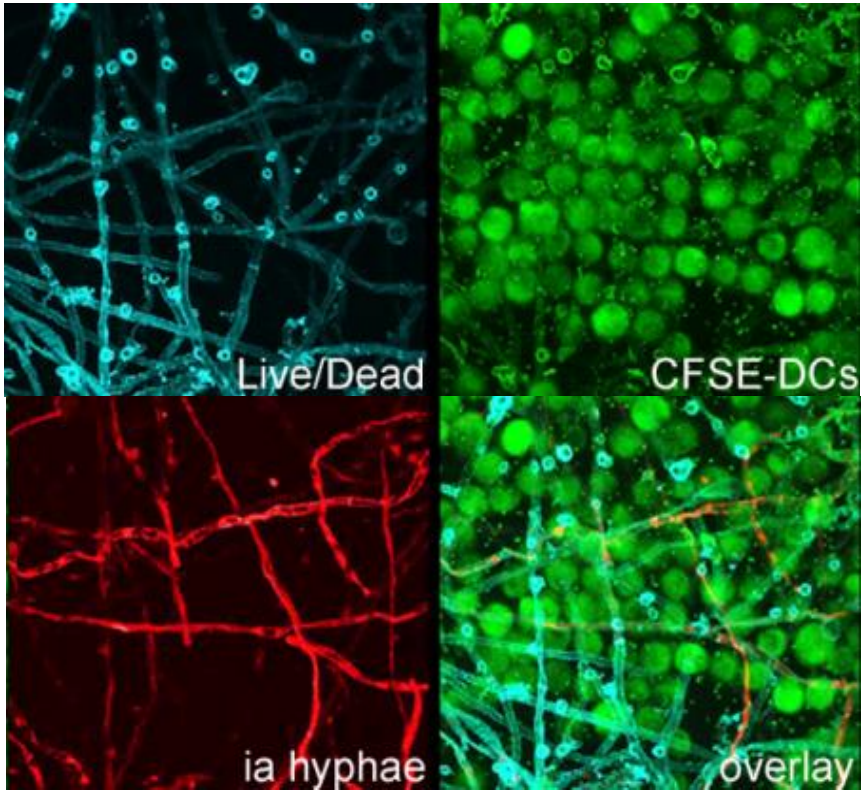

Supplementary Figure S4

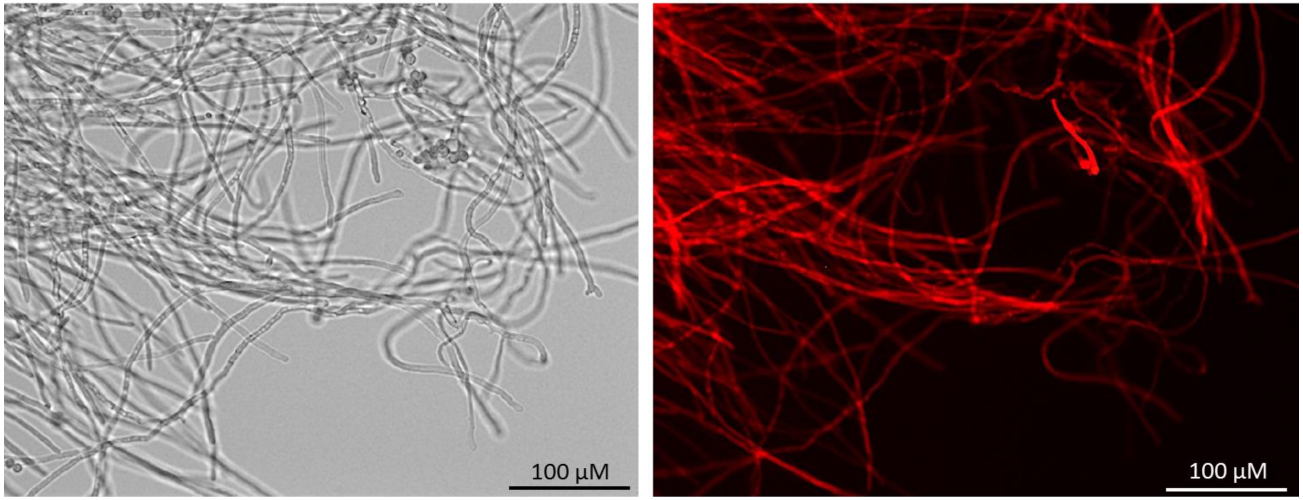

Supplement: Supplementary file 4 — Video Legends and Supplementary Figure Legends and Figures 1 to 4 [file 41598_2017_11271_MOESM4_ESM.pdf]
